# Video-assisted neck surgery (VANS) using a gasless lifting procedure for thyroid and parathyroid diseases: “The VANS method from A to Z”

**DOI:** 10.1007/s00595-019-01908-4

**Published:** 2019-11-14

**Authors:** Kazuo Shimizu, Kazuhide Shimizu, Ritsuko Okamura, Takehito Igarashi, Ryuta Nagaoka, Marie Sanada, Iwao Sugitani, Tatsuya Fukumori, Tetsu Yamada

**Affiliations:** 1 Department of Endocrine Surgery, Nippon Medical School, 1-1-5 Sendagi Bunkyo-ku, Tokyo, 113-8603 Japan; 2 Department of Surgery, Kanaji Hospital, 1-5-6 Nakazato Kita-ku, Tokyo, 114-0004 Japan; 3 Department of Neurosurgery, Tokyo Medical and Dental University, 1-5-45 Yushima, Bunkyo-ku, Tokyo, 113-8510 Japan

**Keywords:** VANS method, Endoscopic thyroidectomy, Minimally invasive thyroid surgery, Gasless lifting method

## Abstract

**Purpose:**

To describe and evaluate our video-assisted neck surgery (VANS) method for thyroid and parathyroid diseases.

**Methods:**

We describe in detail the VANS method for enucleation, lobectomy, total (nearly total) thyroidectomy, and lymph node dissection for malignancy and Graves’ disease. In collaboration with the Japan Society of Endoscopic Surgery (JSES), we evaluated several aspects of this method. The JSES evaluated the method for working-space formation and surgical complications, whereas we examined the learning curve of the surgeons, and the cosmetic satisfaction of the patients and the degree of numbness and pain they experienced. We also asked patients who underwent conventional surgery whether they would have selected VANS had it been available.

**Results:**

The working space for 81.5% of the procedures in Japan was created using the gasless lifting method. The learning curve, considering both blood loss and operating time, decreased after 30 cases. Both factors improved for tumors smaller than 5 cm in diameter. Over 60% of the patients who underwent conventional surgery stated that they would have selected VANS, had it been available. Postoperative pain was worse after conventional surgery than after VANS, but neck numbness after VANS was more frequent than expected.

**Conclusions:**

The VANS method is a feasible, safe, and cost-effective procedure with clear cosmetic advantages over conventional surgery.

## Introduction

**Fig. 1:**
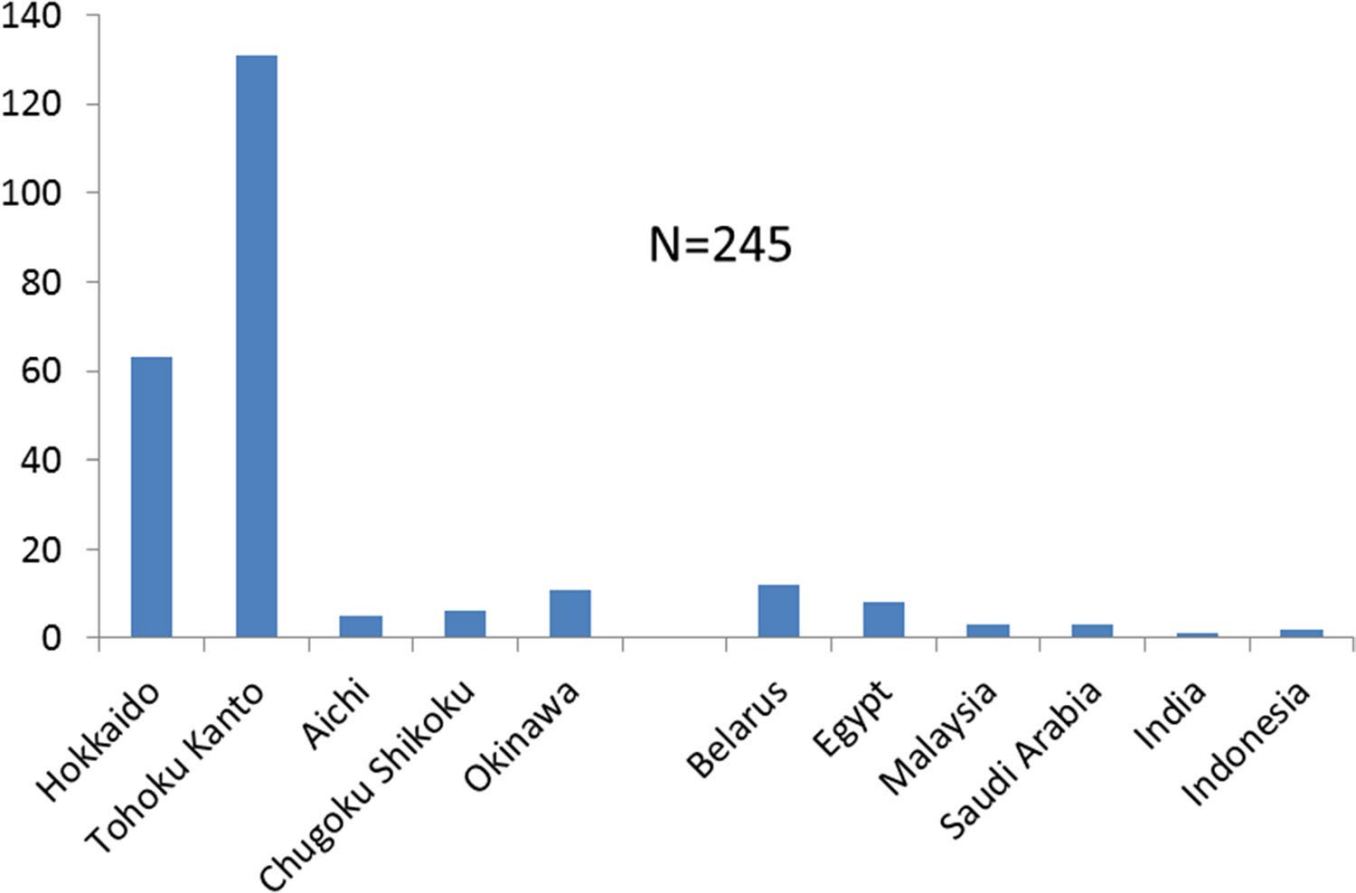
Implementation of the VANS method at other institutes in Japan and other countries. There were 52 cases from Hokuto Hospital, Obihiro, Hokkaido and 119 cases from Kanaji Hospital, Tohoku and Kanto, Therefore, the total number of VANS operations was 935 cases at the time of writing

Cosmetic outcome has always been an important consideration in thyroid and parathyroid surgery because the anterior neck is normally exposed, and thyroid diseases mainly affect women. Thus, thyroid surgeons have sought to improve the cosmetic results of these procedures. To achieve this goal, Gagner et al. [1] described endoscopic parathyroid surgery for secondary hyperparathyroidism in 1996. The first reports of endoscopic thyroid surgery were published by Yeung in Hong Kong [2] and Huscher in Italy [3] in 1997. However, CO_2_ insufflation was used to create the working space, and this can cause surgical complications such as massive emphysema and severe arrhythmia from hypercarbia in the initial period [4]. In 1998, we developed a new procedure for video-assisted endoscopic thyroid and parathyroid surgery, using a gasless skin-lifting method, and named it the “video-assisted neck surgery” (VANS) method [5]. This procedure uses a unique gasless method to create the working space by lifting the skin, creating an open wound without the need for CO_2_ insufflation. Since then, many endoscopic thyroid surgeries have been performed all over the world, greatly influencing the fields of Otolaryngology (head and neck surgery) and endocrine surgery [6–27]. We have published several articles in English [28–34] and Japanese [35] on the VANS technique. In our series, the VANS method was used to treat 690 patients with various thyroid and parathyroid diseases from 1998 to 2014, which represented 23% of the endocrine neck surgeries performed at our institute in this period (Table 1). The VANS method has been performed at other institutes in Japan and in different countries (Fig. 1), bringing the total number of these procedures to date close to 950.

**Table 1:** Percentages of endocrine neck operations performed using the video-assisted neck surgery (VANS) method at Nippon Medical School (March 1, 1998–March 31, 2014)

Disease	Total cases	VANS	%
Thyroid	2634	671	25.5
Benign	1159	520	44.9
Malignant	1070	116	10.8
Grave’s disease	372	34	9.1
Chronic Thyroiditis	33	1	3.0
Parathyroid	298	18	5.9
PHP	261	17	6.5
SHP	43	1	2.3
Others	73	1	1.4
Total	3005	690	23.0

*PHP* primary hyperparathyroidism, *SHP* secondary hyperparathyroidism

In April 2016, the Japanese government approved insurance coverage of endoscopic thyroid surgery for benign nodules and Graves’ disease. This decision renewed interest in endoscopic thyroid surgery throughout Japan because of its cosmetic benefit. In 2018, insurance coverage of endoscopic thyroid surgery for treating malignant tumors was also approved. These approvals prompted surgeons to continue developing endoscopic thyroid surgical techniques [36, 37].

**Fig. 2:**
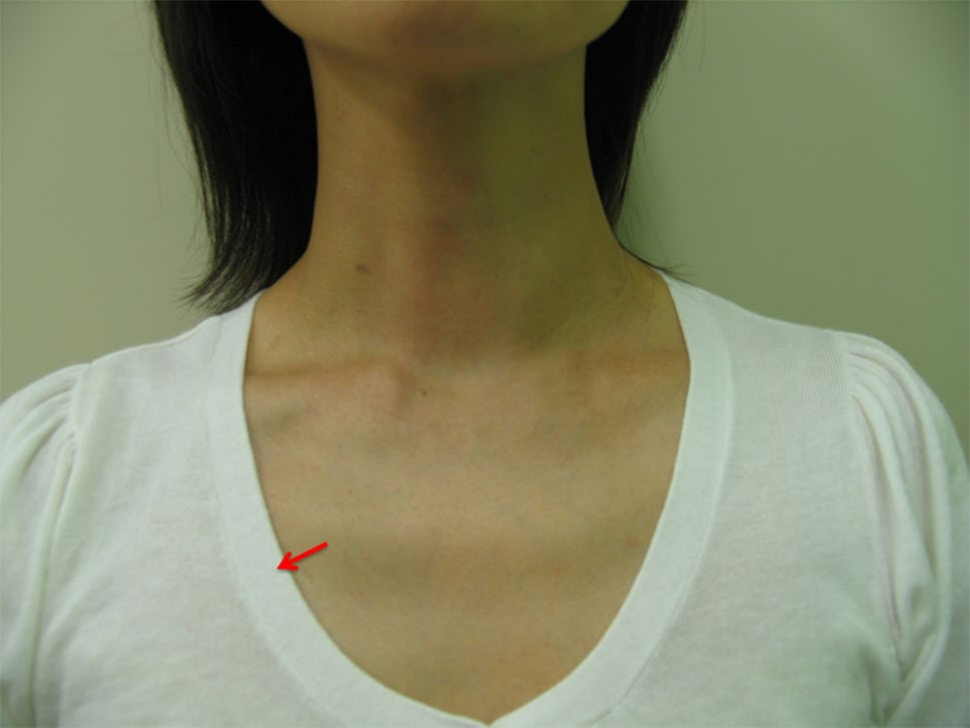
A patient who underwent thyroid surgery by the VANS method almost 1 year earlier. The main chest wound is indicated by the red arrow

We describe, step-by-step, how to perform these procedures using the VANS method for endocrine surgeons who wish to learn and apply them, including the skin incision, working-space formation, approach to the thyroid gland, resection of thyroid lesions, and wound closure. Following this, we review studies evaluating the VANS method, which support that it achieves surgical results comparable to those of conventional surgery, with excellent cosmetic outcomes (Fig. 2).

## Methods

The research protocol was conducted according to the principles documented in the Declaration of Helsinki and approved by the local ethics committees of Nippon Medical School. The requirement for signed informed consent was waived due to the retrospective nature of this study.

First, we describe the following four steps of the VANS method in detail: setup, incision and working-space, application in various thyroid diseases, and wound closure. Then we evaluate the various aspects of the VANS method through a number of surveys conducted by us, and also by the Japan Society of Endoscopic Surgery (JSES). This included JSES questionnaires on the lifting method; a JSES questionnaire on endoscopic thyroid/parathyroid surgery for different disease types; a JSES questionnaire on surgical complications; several related abstracts from major congresses in Japan; the learning curve [29] [reprinted from Best Pract Res Clin Endocrinol Metab, 15, Shimizu K, Minimally invasive thyroid surgery, 123–137, Copyright (2001), with permission from Elsevier]; the effect of tumor size on operating time and blood loss [35] [reprinted from Nihon Gekagakkai Zasshi 2002; 103(10)]; a questionnaire on wound satisfaction; a questionnaire on postoperative numbness of the neck; a questionnaire on postoperative pain; a questionnaire on choice of surgical procedure; and comparison of the VANS method in Belarus and Japan. Corresponding results are described according to these surveys.

### Set up for the VANS method

**Fig. 3:**
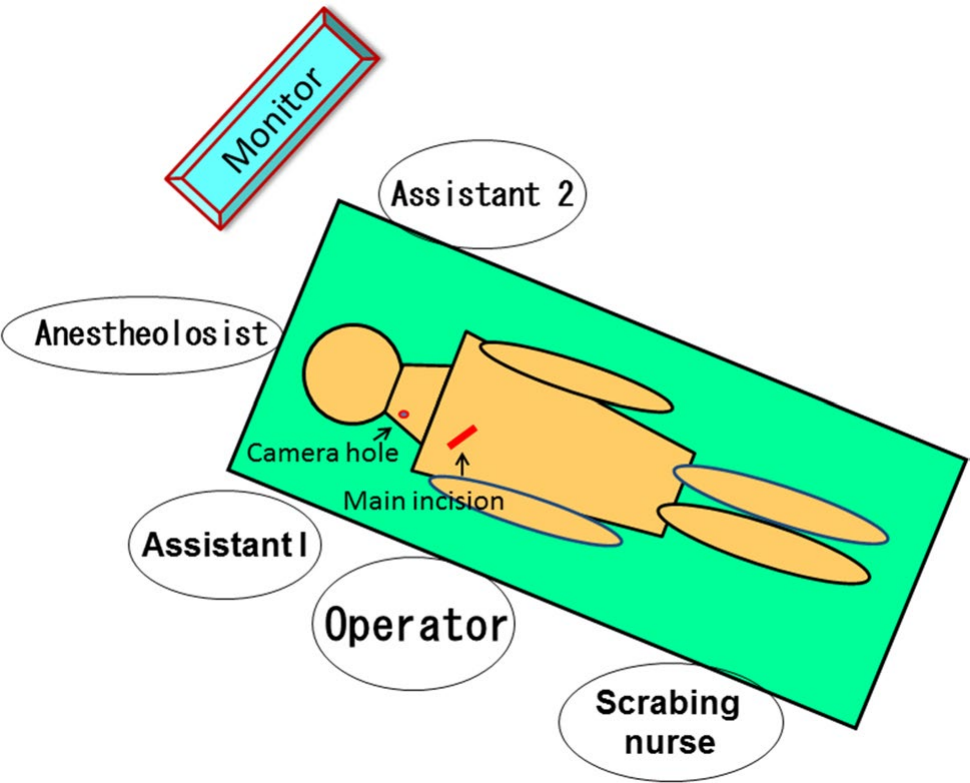
Positioning of the operative team. This patient has a tumor in the right side of the neck

Under general anesthesia, with the patient lying on their back, slight extension of the anterior neck is achieved by placing a pillow under the shoulders. The patient’s arms are positioned by their side, close to the trunk. The operator stands on the side of the tumor. The first assistant, who holds the camera, is on the same side as the operator, closer to the patient’s head. The scrub nurse is also located on the same side as the operator, caudally. The second assistant, if needed, stands on the patient’s other side to assist the operator with suction, muscle retraction, and so on. The anesthesiologist is always beyond the patient’s head area. The monitor is positioned where it can be viewed comfortably and easily by the operator (Fig. 3).

### Incision and working-space creation

First, the anterior neck region is infiltrated with 20 mL of saline containing 0.6% epinephrine to prevent unnecessary bleeding. A small main oblique incision, 2.5–4.5 cm long, depending on the tumor size, is made in the tumor side of the chest wall, in a region that is normally concealed by open-, V- or U-neck clothing. From this wound, the layer under the platysma in the anterior neck is dissected using an electrosurgical or ultrasonically activated scalpel (UAS).

**Fig. 4:**
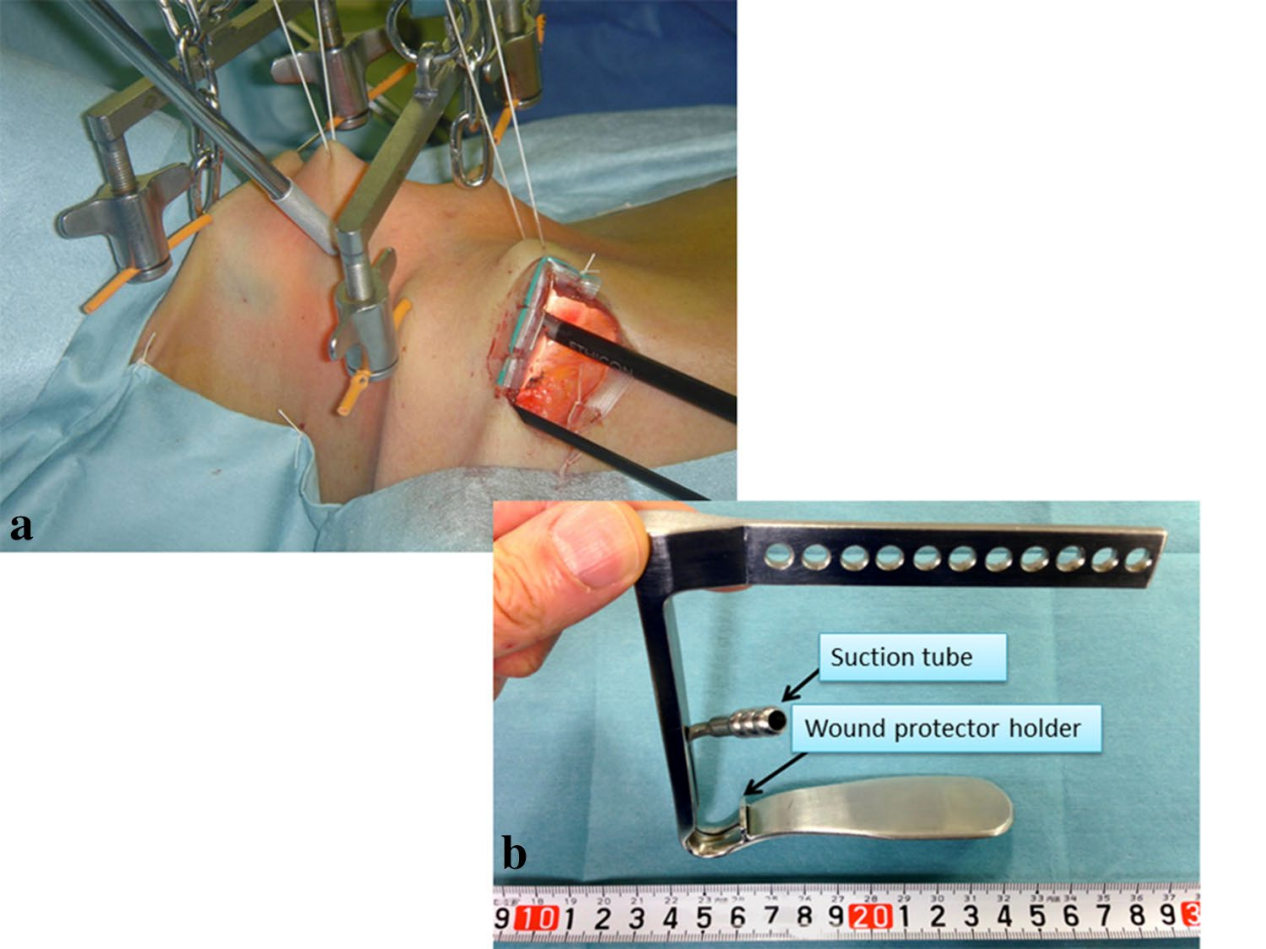
Overall view of the VANS method and recent retractor. Left: original VANS method. The main incision is made in the anterior chest wall of the tumor side and a 5 mm camera port is inserted from the same side. Two pieces of Kirschner wire are connected to two handles, which are pulled up by chains connected to a reversed L-shaped bar. A 5-mm camera port is placed in the right lateral neck. Right: a mistless new retractor, donated by Dr. Katayama, was recently introduced. The operative field is always kept clear by continuous suctioning from a tube connected to the retractor. The retractor is lifted up using one or two optimal holes

To obtain the working space, two pieces of 3-mm-diameter Kirschner wire are inserted horizontally and subcutaneously. This subcutaneous insertion technique prevents injury to the anterior jugular veins. These wires are then lifted by two chains with handles that are connected to a reversed L-shaped bar, which is fixed to the operating table near the other side of the patient’s head. The center of the anterior neck is also lifted up like a tent with suture material, to achieve a wider space. The skin edge of the operating wound is covered with silicon to protect the patient from mechanical injury caused by insertion of the instruments. A 5-mm endoscope is then inserted from the same side of the lateral neck through a scope guide, to project an image of the operative field (Fig. 4a).

Instead of lifting with pieces of wire, a mistless retractor connected to a suction tube [25]) was recently implemented to create the working space (Fig. 4b). Continuous suction during the operation enables the operator to observe the operating area more clearly. A 3-mm-diameter grasper and UAS are then inserted through the main chest wound. The approach to the thyroid depends on the lobe in which the lesion is located. The 5-mm camera is inserted from the lateral neck to the 1/3 caudal position between the two wires in the original method, and at the 1-cm lateral position of the mistless retractor in the modified method.

## Application of the VANS method for various thyroid diseases

### Enucleation

This method is used mainly for benign lesions, less than 3 cm in diameter, located in the anterior part of the thyroid. If the lesion of interest is located in the right hemi-lobe, a right lateral approach is recommended. After a sufficiently wide working space is obtained, the first incision is made along the right medial margin of the sternocleidomastoid muscle from caudal to cephalad until the right omohyoid muscle is identified. The inner margin of the omohyoid muscle and the lateral margin of the sternohyoid muscle are then split up to the hyoid bone. The lateral margin of the sternohyoid muscle is dissected further caudally to the right clavicle, exposing the triangle created by the right sternocleidomastoid, the right omohyoid, and the right sternohyoid muscles. The sternothyroid muscle, which directly covers the thyroid, can be split, displaced, or cut, depending on the tumor size. If the tumor margin is clear, dissection can be conducted along the tumor capsule without needing to identify the recurrent nerve or the parathyroid.

### Lobectomy

If a tumor is located on the right side, the right superior thyroid artery is dissected first, but after the bifurcation site of the antero-medial and the posteo-lateral branches. This is to prevent injury to the external branch of the superior laryngeal nerve, which runs along the main trunk of the superior thyroid artery, although the situation differs from case to case. When dissecting this area to the trachea, the right superior parathyroid comes into view. Preservation of a small portion of the normal thyroid at the superior level is also helpful to protect against right recurrent nerve injury. The superior thyroid veins are dissected in the same manner as the artery. The use of a UAS is safe enough to achieve hemostasis without ligation or clipping. The lower pole of the thyroid is then mobilized to expose the anterior portion of the trachea, which is a safe zone because it does not contain important nerves or vessels. At this point in the operation, the operator can see the recurrent nerve, the inferior thyroid artery, and the lower parathyroid on the image.

The recurrent nerve runs behind Zuckerkandl’s tubercle just before it enters the cricoid cartilage at Berry’s ligament. It also runs rectangularly to cross the inferior thyroid artery anteriorly or posteriorly and comes up from behind the subclavian artery. Therefore, the right recurrent nerve is easy to identify if the operator understands these three anatomical relationships. The left recurrent nerve is located slightly closer to the trachea than the right nerve, but runs in almost the same direction.

An important step is dissection of the thyroid from Berry’s ligament at the site where the recurrent nerve enters the cricoid cartilage. The recurrent nerve is close to the thyroid at this point and small vessels run through this area. The active blade of the UAS should be at least 3 mm away from the nerve to prevent direct thermal stimulation. Frequent cooling with cold fluid is recommended while using the UAS.

The superior parathyroid is located mainly around the entrance of the recurrent nerve at the level of the cricoid cartilage. Therefore, when a portion of the superior thyroid is dissected, the right superior parathyroid naturally comes into view. Compared with the superior parathyroid, the inferior parathyroid is located mainly in the fatty tissue attached to the normal lower thyroid lobe. It is a darker yellow than normal fatty tissue and capillary arteries are sometimes seen on its superior surface. The location of the inferior parathyroid has more anatomical variety than that of the superior parathyroid, such as in the thymic tongue, carotid sheath, next to the esophagus, or in the mediastinum. Finally, the mobilized right lobe is dissected from the anterior portion of the trachea. Left lobectomy is performed using the same procedure as described for the right lobectomy, with a left main incision and approaching from the left.

### Total (nearly total) thyroidectomy

If the lesion is located in both lobes or if the operative manipulation needs to involve both lobes (total or nearly total thyroidectomy), such as for multinodular goiter, Graves’ disease, or thyroid cancer, a central approach is used. The linea alba of the sternohyoid muscle is split from the sternal notch to the upper border of the thyroid cartilage. The sternothyroid muscle is also split from the thyroid laterally to expose the whole thyroid.

After performing right lobectomy as described above, the dissection moves to the left lobe. First, the patient’s face is moved a little to the right. After displacing the left sternothyroid muscle laterally (it can be cut, if necessary), dissection should start at the left superior pole in the same manner as described for the right lobe. If the left superior pole of the thyroid is difficult to identify, the end of the sternothyroid and the sternohyoid muscles attached to the hyoid bone can be cut laterally. The edge of the isthmus is grasped by clamps and pulled to the right to expose the whole left lobe. Since the sternothyroid and sternohyoid muscles are displaced laterally, dissection of the left superior pole in the thyroid manipulation is relatively easy. In the same manner as for right lobe resection, mobilization is done from the superior to the inferior of the thyroid until identification of the left recurrent nerve, which is located at the tracheo-esophageal sulcus. If it is difficult to identify the left recurrent nerve, inserting an endoscope from the left lateral neck through a 5-mm incision is strongly recommended. The left recurrent nerve comes into view in the fatty tissue at the tracheo-esophageal sulcus. The left parathyroids are identified in almost the same way as the right parathyroids.

### Lymph node dissection in thyroid cancer

The operative indication for thyroid cancer is strictly limited when not performed by conventional surgery. The operative procedure differs from lobectomy to total thyroidectomy, depending on the tumor size and location. The indication for using endoscopic surgery to treat malignancy is described elsewhere [29–33]. Briefly, a papillary carcinoma less than 2 cm in diameter with no lymph node swelling suggesting metastasis, or a genetically positive familiar medullary carcinoma of the thyroid (MCT) without any clinical findings of tumor development, is a suitable indication for lobectomy or total thyroidectomy.

If lymph node dissection is needed, it is important that the UAS is never used within 3 mm of the recurrent nerve. Alternatively, the use of fine scissors is recommended to prevent heat injury to the nerve. It is possible to create a lateral compartment as well as a central compartment, paying careful attention to the thyrocervical artery and vein, accessory nerves, phrenic nerve, and thoracic duct, which are identified when dissecting the left jugular angle. To prevent injury to these structures, do not dissect deeper than the thyrocervical artery level. Dissection of the central zone should be performed along the recurrent nerve in the area inside the carotid artery to the upper limit of the thyroid cartilage.

### Operative procedure for Graves’ disease

The operative procedure for Graves’ disease is total thyroidectomy or nearly total thyroidectomy to prevent relapse. For this reason, the total amount of remnant thyroid should be less than 2 g. The size limit of the thyroid gland for endoscopic surgery is approximately 100 g, since the thyroid in this disease tends to bleed easily. Preoperative iodine treatment is recommended to reduce vascularity and bleeding during the operation. Therefore, the operative procedure is more delicate than those for other thyroid diseases. As described for total or nearly total thyroidectomy, thyroidectomy for Graves’ disease is performed from right lobectomy to left lobectomy. When performing left lobectomy, the insertion of an endoscope from the left lateral side of the neck is useful for imaging the left recurrent nerve and the parathyroid. If the left recurrent nerve is difficult to see, even by inserting the endoscope from the left side, a one- or two-step resection of the left lobe is helpful to identify the left recurrent nerve and the left parathyroids. If nearly total thyroidectomy is performed, a small amount of remnant thyroid should be preserved near the bilateral entrance of the recurrent nerve to the cricoid cartilage.

### Closure of the wound

Before closing the wound, the pillow is removed so that the patient is returned to a natural position, to check for any minor bleeding after loosening the tonus of the anterior muscles and other anterior areas. Hemostasis is confirmed by applying air pressure of 25–30 mmHg. With the lateral approach, the split sternohyoid and omohyoid muscles are sutured once or twice. With the central approach, the split sternohyoid muscles are sutured several times and closed. Generally, a drain is not placed, although if needed, a 3-mm soft drain is placed in the area of resection, from the lateral edge of the main wound without making a new wound.

## Results

### Evaluations of the VANS method

1. Survey performed by the JSES in 2016 evaluating the method used to create the working space in endocrine neck surgery (*N* = 3262).

**Fig. 5:**
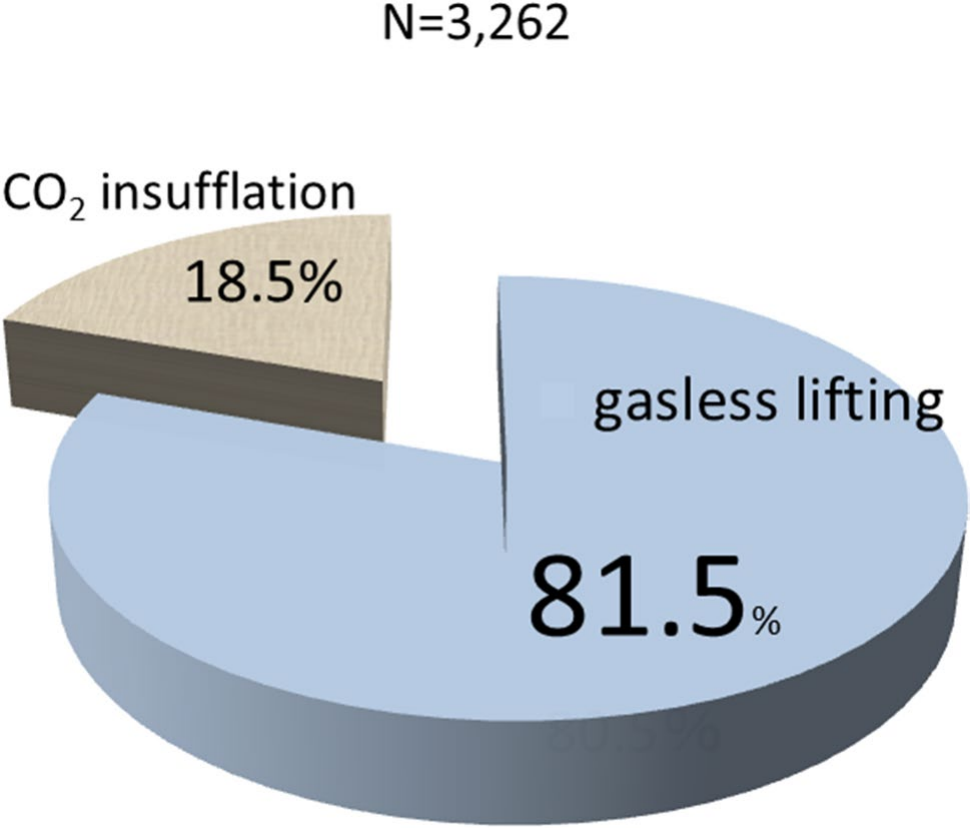
Results of the questionnaire on working space creation between the gasless lifting method and the gas insufflation method. The gasless lifting procedure creates 81.5% of the working space (reported by the JSES)

Figure 5 shows the results of this JSES questionnaire comparing the gasless lifting method with the gas insufflation method to create the working space in endoscopic endocrine neck surgery. The gasless lifting method was used more often, accounting for 81.5% of the procedures in Japan.

2. Results of the JSES questionnaire on thyroid and parathyroid diseases treated endoscopically in Japan (*N* = 3262).

**Fig. 6:**
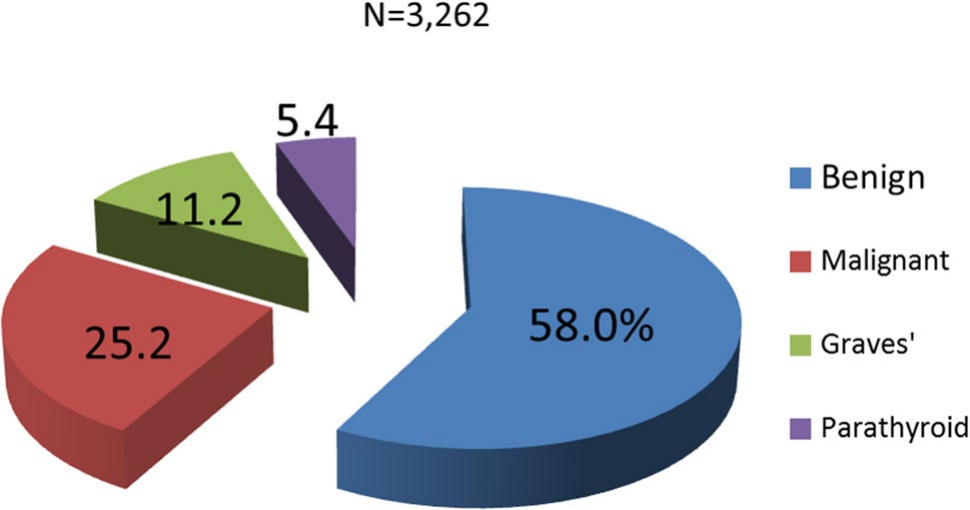
Various thyroid diseases extirpated by endoscopic thyroid surgery (reported by JSES). Benign thyroid disease was most common, followed by malignant thyroid disease and Graves’ disease. The percentages of thyroid diseases are almost same as in our results

Figure 6 shows the percentages of various thyroid and parathyroid diseases treated by endoscopic surgery according to the JSES questionnaire. Benign thyroid diseases were the most common, followed by malignant diseases, Graves’ disease, and parathyroid disease. These results were almost the same as ours (Table 1)

3. Results of the JSES questionnaire on surgical complications (*N* = 3262).

**Fig. 7:**
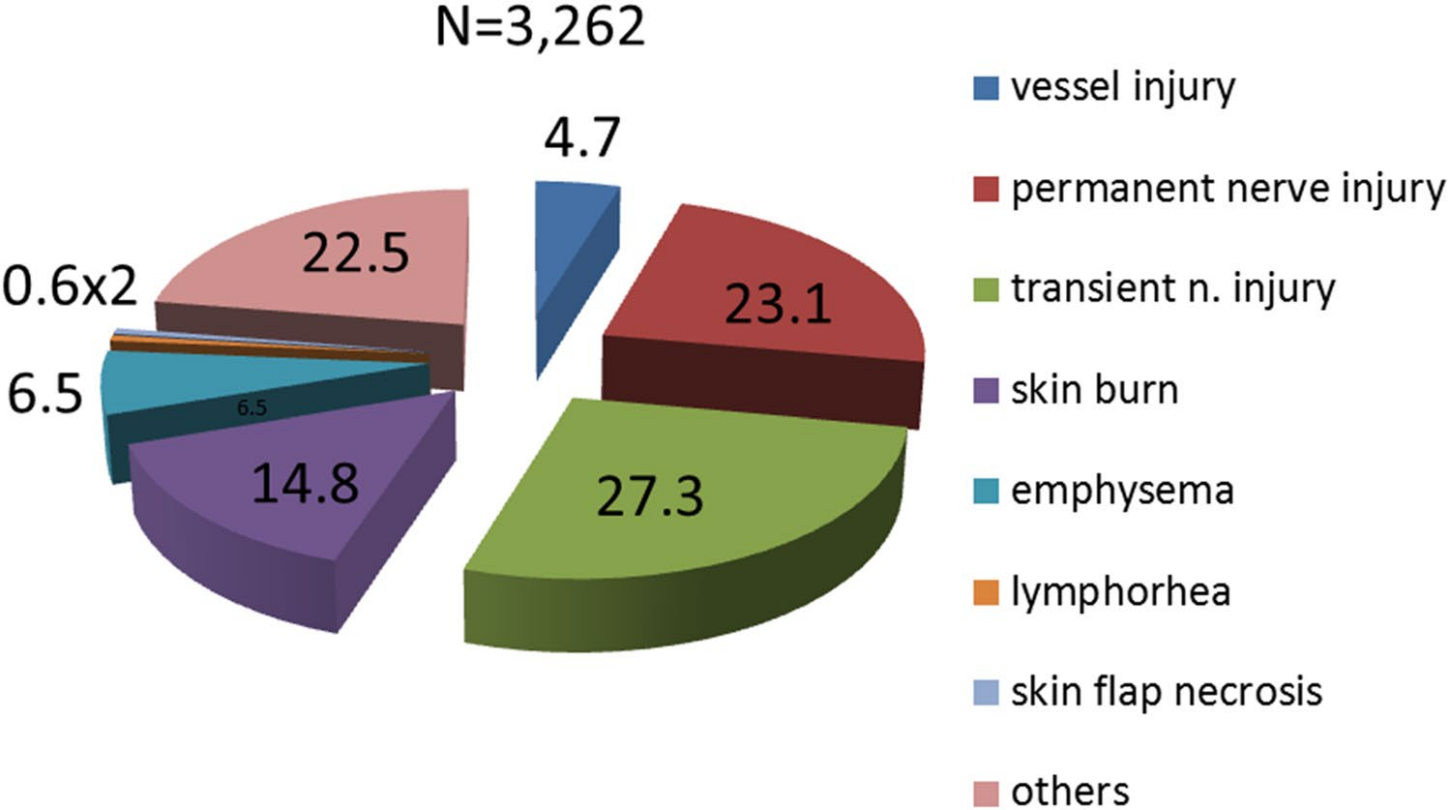
Complications of VANS. Transient and permanent nerve injury was the most common, followed by skin burn, emphysema, and vessel injuries in that order (Reported by JSES). Skin burns were more common than in our results

Figure 7 shows the surgical complications after endoscopic thyroid surgery, according to the JSES questionnaire. The total number of permanent and transient nerve injuries accounted for 50.4% of the complications, followed by skin burn (14.8%), emphysema (6.5%), and vessel injury (4.7%). Our complication results were almost the same as those of the JSES except for the skin burn and emphysema.

4. Number of abstracts reported in three congresses from 1998 to 2018 in Japan.

**Fig. 8:**
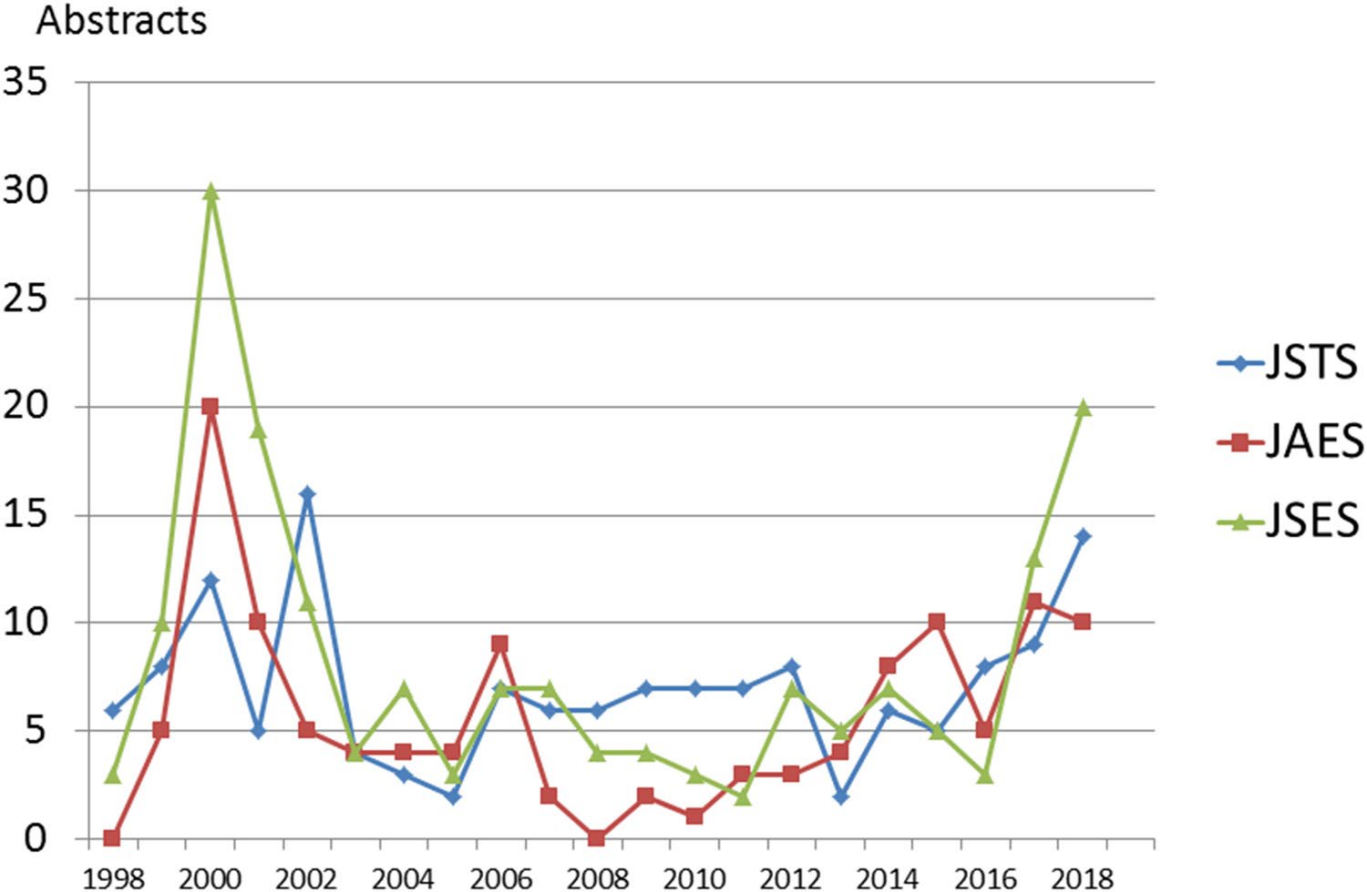
Number of abstracts on endoscopic thyroid and parathyroid surgery presented at three major affiliated congresses in Japan. In 2000, the number of endoscopic thyroid operations increased sharply and then gradually decreased until again increasing after insurance was approved for benign nodules and Graves’ diseases in 2016 and malignant thyroid diseases in 2018

Figure 8 shows the number of abstracts included in the three major congresses related to endoscopic thyroid and parathyroid surgery between 1998 and 2018 in Japan. The number of abstracts increased sharply in 2000, and after a temporary increase in 2007, leveled off, and then again began to increase from 2017 onwards. Overall, after insurance coverage approval of endoscopic surgical treatments in Japan, more abstracts on these thyroid diseases were published within that same year. On the other hand, after 2000, the number of abstracts on parathyroid diseases gradually decreased and remained low thereafter. Recent improvements in the technology for locating pathological parathyroid lesions have made parathyroidectomy easier to perform without an endoscopic technique, but rather by conventional surgery with a small (2*–*3-cm) incision in the neck (data not shown).

5. Learning curve for the VANS method.

**Fig. 9:**
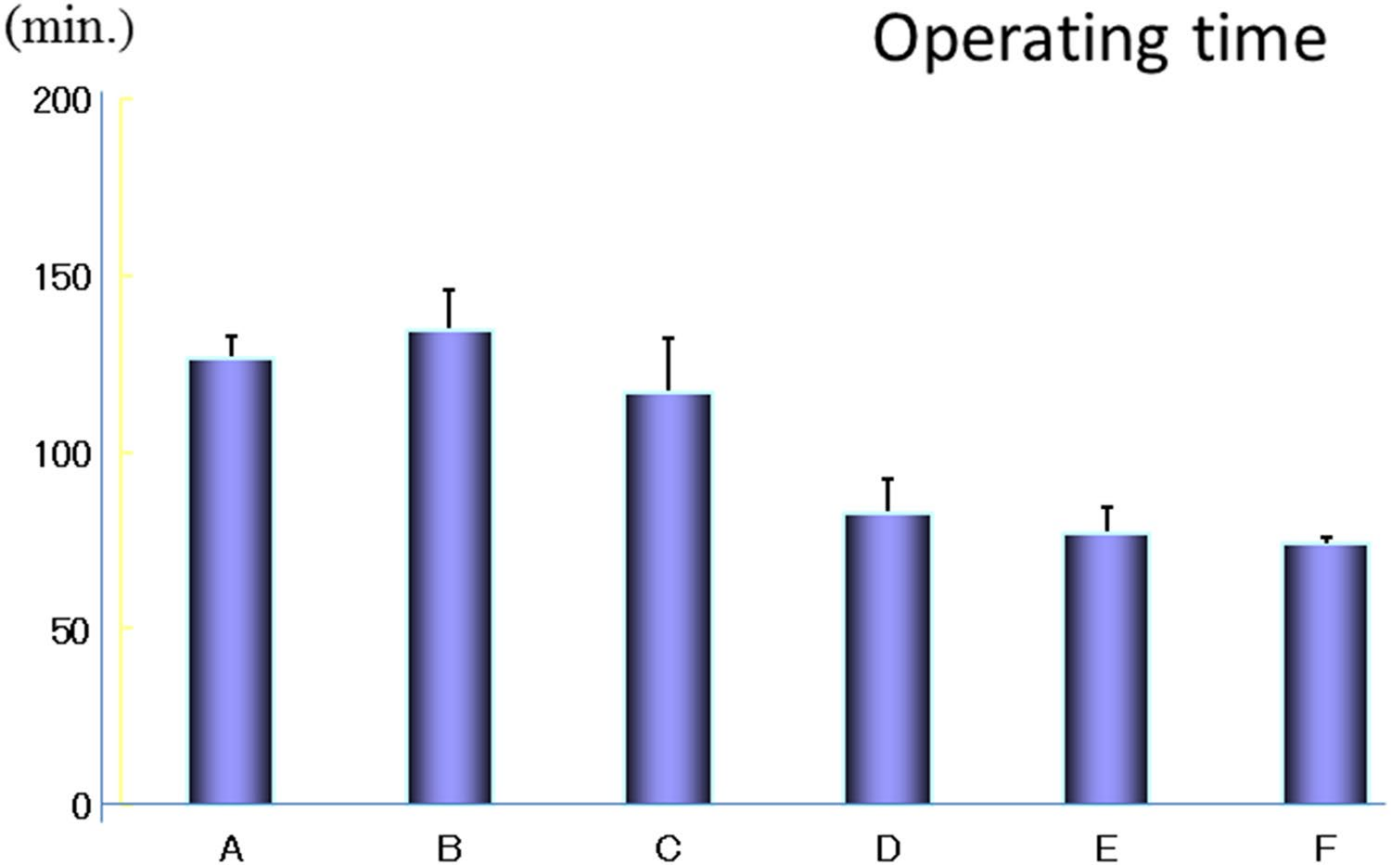
The learning curve for the VANS method according to the operating time in 60 cases performed by a single surgeon. These 60 cases were divided into 6 groups of 10 from A to F. The operating time decreased remarkably after 30 cases. Reprinted from Best Pract Res Clin Endocrinol Metab, 15, Shimizu K, Minimally invasive thyroid surgery, 123–137, Copyright (2001), with permission from Elsevier [29]

**Fig. 10:**
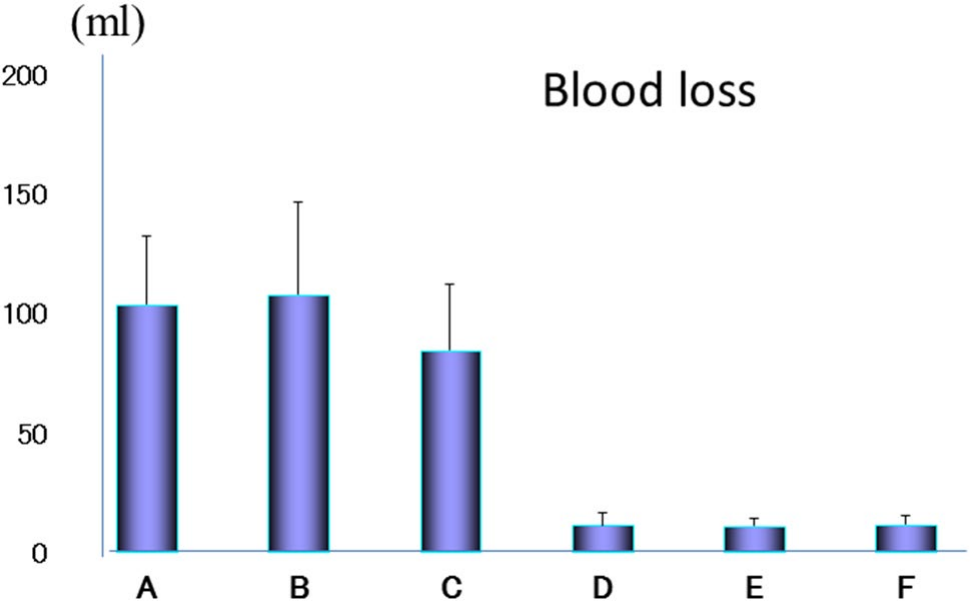
The learning curve for the VANS method according to the blood loss in 60 cases performed by a single surgeon. The blood loss also decreased remarkably after 30 cases in the same manner. Reprinted from Best Pract Res Clin Endocrinol Metab, 15, Shimizu K, Minimally invasive thyroid surgery, 123–137, Copyright (2001), with permission from Elsevier [29]

Figures 9 and 10 show the learning curves assessed by operating time and blood loss [29]. Sixty continuous cases treated by a single surgeon, starting with the first case, were divided into six groups from A (earliest) to F (latest). For each group of ten cases, the operating time and blood loss were measured. Both of these factors decreased remarkably after 30 cases of continuous experience by the surgeon.

6. Effect of tumor size on the operating time and blood loss in the VANS method.

**Fig. 11:**
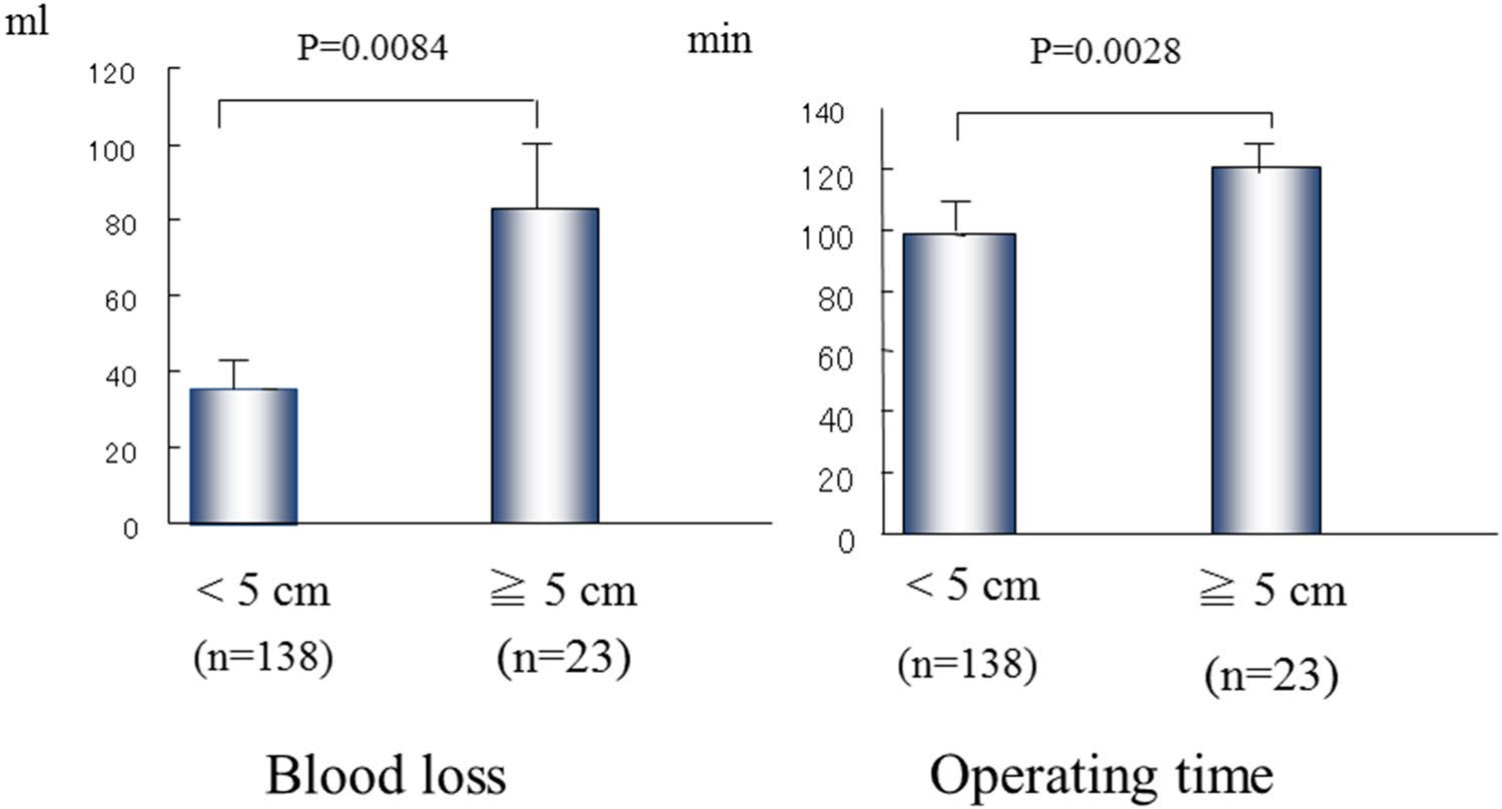
Statistical analysis of tumor size in relation to blood loss and operating time. Tumors smaller than 5 cm are most suitable for the VANS method at present. Reprinted from Nihon Gekagakkai Zasshi 2002; 103(10) [35])

In general, the tumor size had a marked effect on the operating time and blood loss. Tumors of several sizes were investigated for both factors. A significant difference was found for tumors < 5 cm versus those ≧5 cm in diameter, at *p* = 0.0084 for blood loss and *p* = 0.0028 for operating time (Fig. 11) [35]. The operating time and blood loss were obviously increased for tumors over 5 cm.

7. Results of a questionnaire on wound satisfaction by age bracket in patients undergoing the VANS method or conventional surgery.

**Fig. 12:**
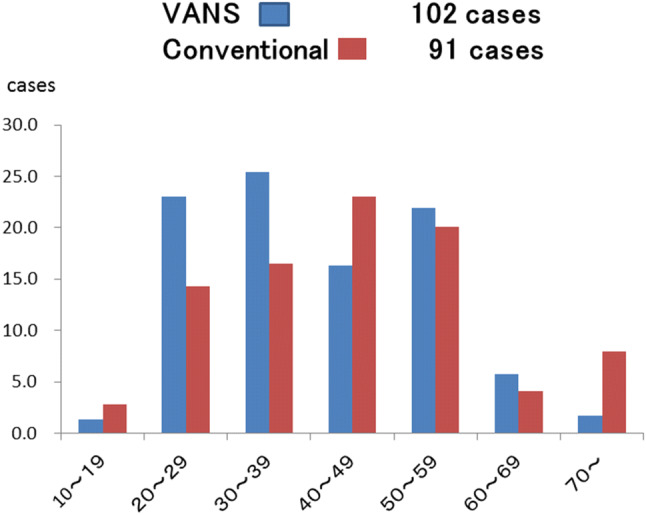
Questionnaire about cosmetic satisfaction after the VANS method and conventional surgery by age bracket. Patients in the 10–19, 40–49, and over 70 year age brackets reported a cosmetic advantage after conventional surgery contrary to expectation. On the other hand, more patients in the 20–39 and 50–69 year age brackets expressed satisfaction after the VANS method

The degree of postoperative wound satisfaction by age brackets of 10 years each, from 10 to over 70 years old, was studied. The postoperative period was randomly investigated. Although most of the patients were 20–59 years of age, those in the 20–39 years age bracket expressed greater wound satisfaction with the VANS than the conventional method. In contrast, patients in the 10–19, 40–49, and over 70 years age brackets expressed greater satisfaction with conventional surgery (Fig. 12). The possible reasons for this are that some of the youngest patients might not have understood the difference, while those over 70 years of age might not have cared about the postoperative wound appearance. We do not understand why the patients 40–49 years of age expressed more satisfaction about the conventional operation scar. However, we suspect that they were unaware of the scar appearance from the VANS procedure, for comparison.

8. Results of a questionnaire on postoperative numbness of the neck between the VANS method and conventional surgery.

**Fig. 13:**
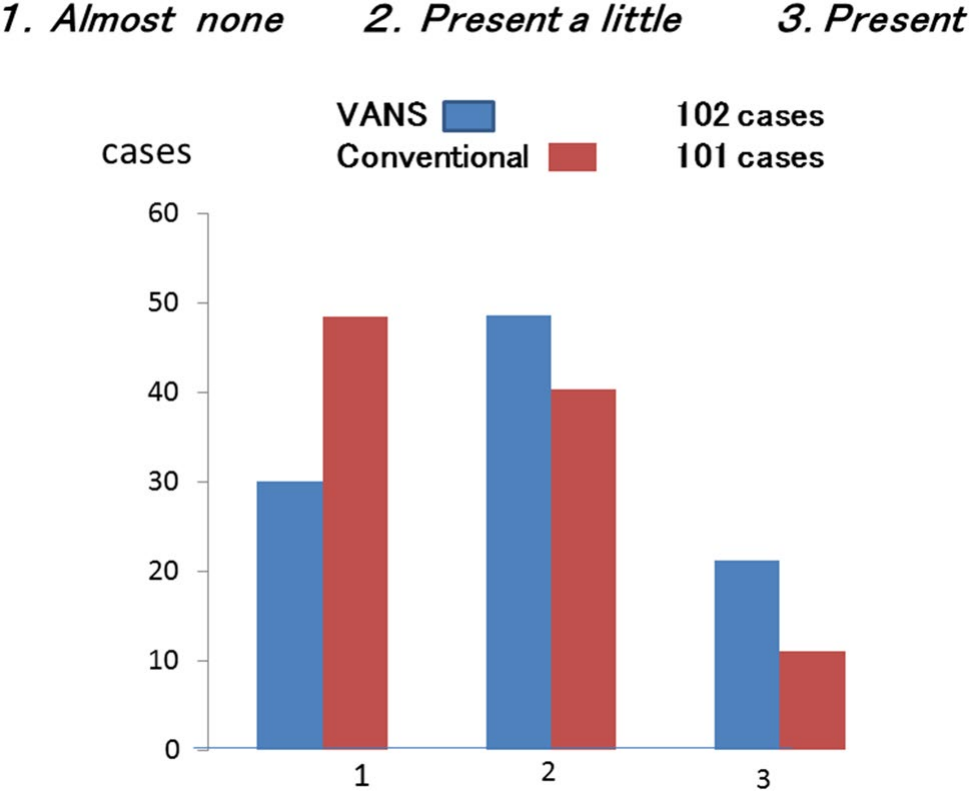
Questionnaire on numbness of the anterior neck within 1 year of the VANS method versus conventional surgery. (1) Immediately after the operation, (2) less than 6 months after the operation, (3) 7–12 months after the operation

Unexpectedly, numbness was experienced by more VANS patients than by conventional surgery patients (Fig. 13). This was probably because a wider dissection is made in the VANS method than in conventional surgery to obtain the working space. However, most of the discomfort typified by numbness had disappeared within 1 year.

9. Results of a questionnaire on postoperative pain after the VANS method versus conventional surgery.

**Fig. 14:**
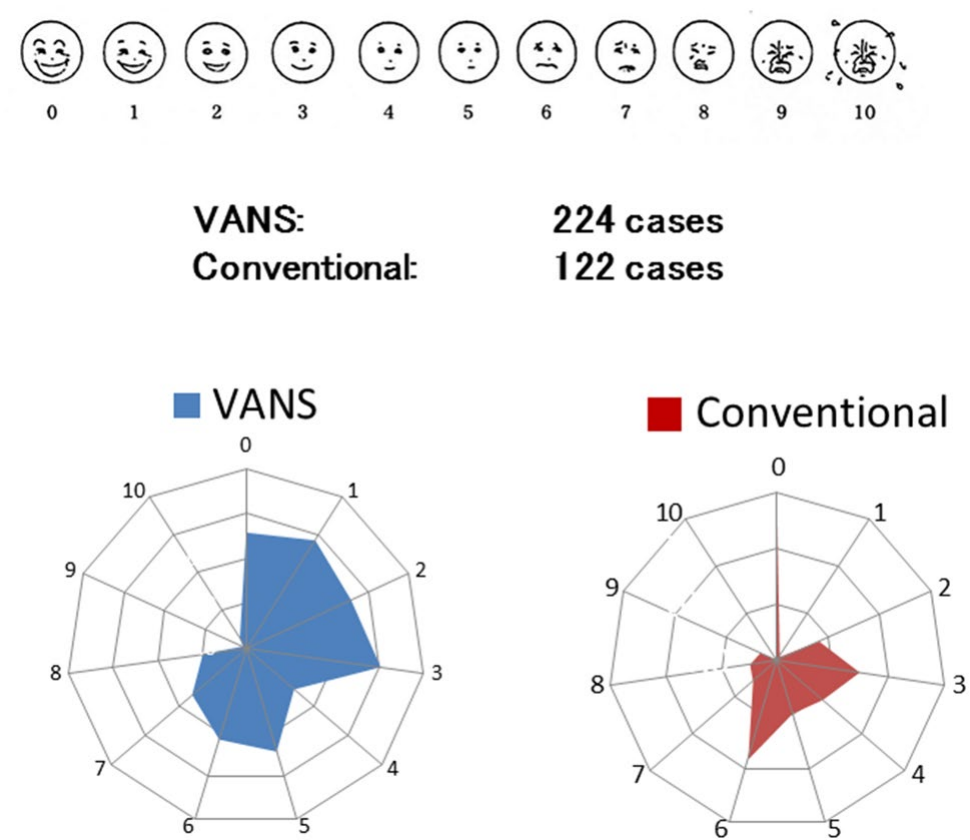
Questionnaire on postoperative pain within 1 year. Most of the patients who underwent the VANS method reported only slight pain whereas those who underwent a conventional surgery reported worse pain

Postoperative pain within 1 year after surgery was rated by patients as 0 (no pain) to 10 (unbearable pain). Most of the patients who underwent the VANS method rated their postoperative pain in the low range of 0–3, whereas those who underwent conventional surgery tended to rate their postoperative pain in the higher range of 3–6 (Fig. 14). The pain after the VANS method also appeared to decrease year by year compared with that after conventional surgery (data not shown).

10. Patients who underwent conventional surgery were asked if they would have selected endoscopic surgery if the VANS method had been available at the time.

**Fig. 15:**
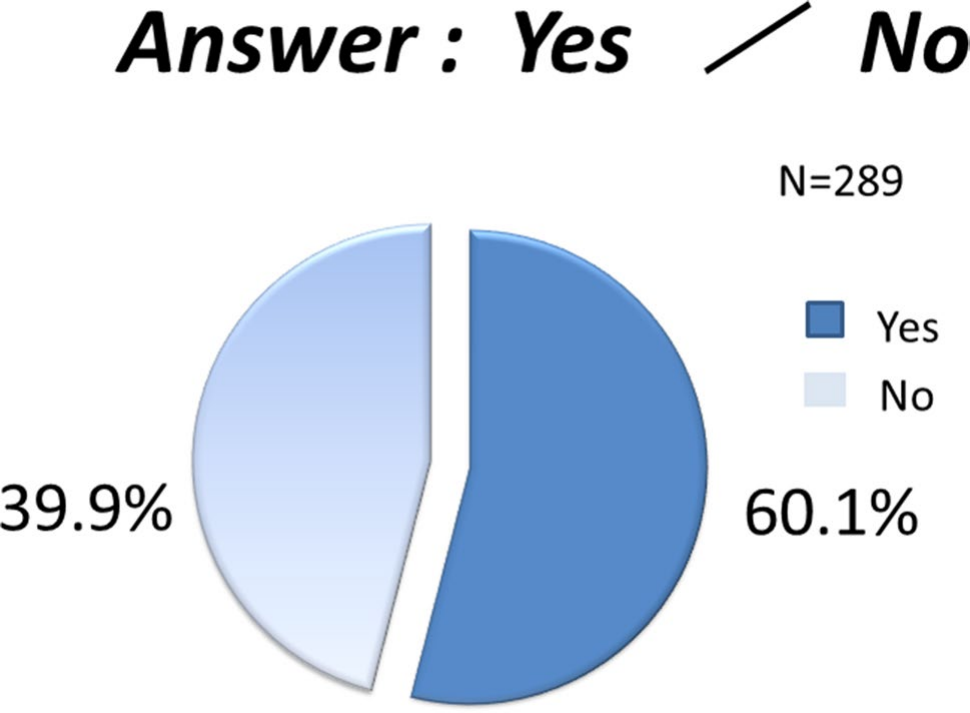
Responses to the question: “Would you have elected to undergo the VANS method of surgery if it had been available when you underwent the conventional operation?” More than 60% of the patients who underwent conventional surgery said that they would have chosen the VANS method

More than 60% of the patients who underwent conventional surgery said they would have selected endoscopic surgery had it been available, even though government insurance coverage was not approved for thyroid or parathyroid diseases at the time. The main reason appeared to be the cosmetic advantage, followed by less pain and discomfort. The high percentage of patients choosing conventional surgery (39.9%) was probably because they were not aware of the cosmetic advantage of endoscopic surgery in the neck at that time. Furthermore, many of these patients were already satisfied with their conventional surgery results (Fig. 15).

11. Comparative study of blood loss and operation time with the VANS method: Belarus (*n* = 9) versus Japan (*n* = 33).

**Fig. 16:**
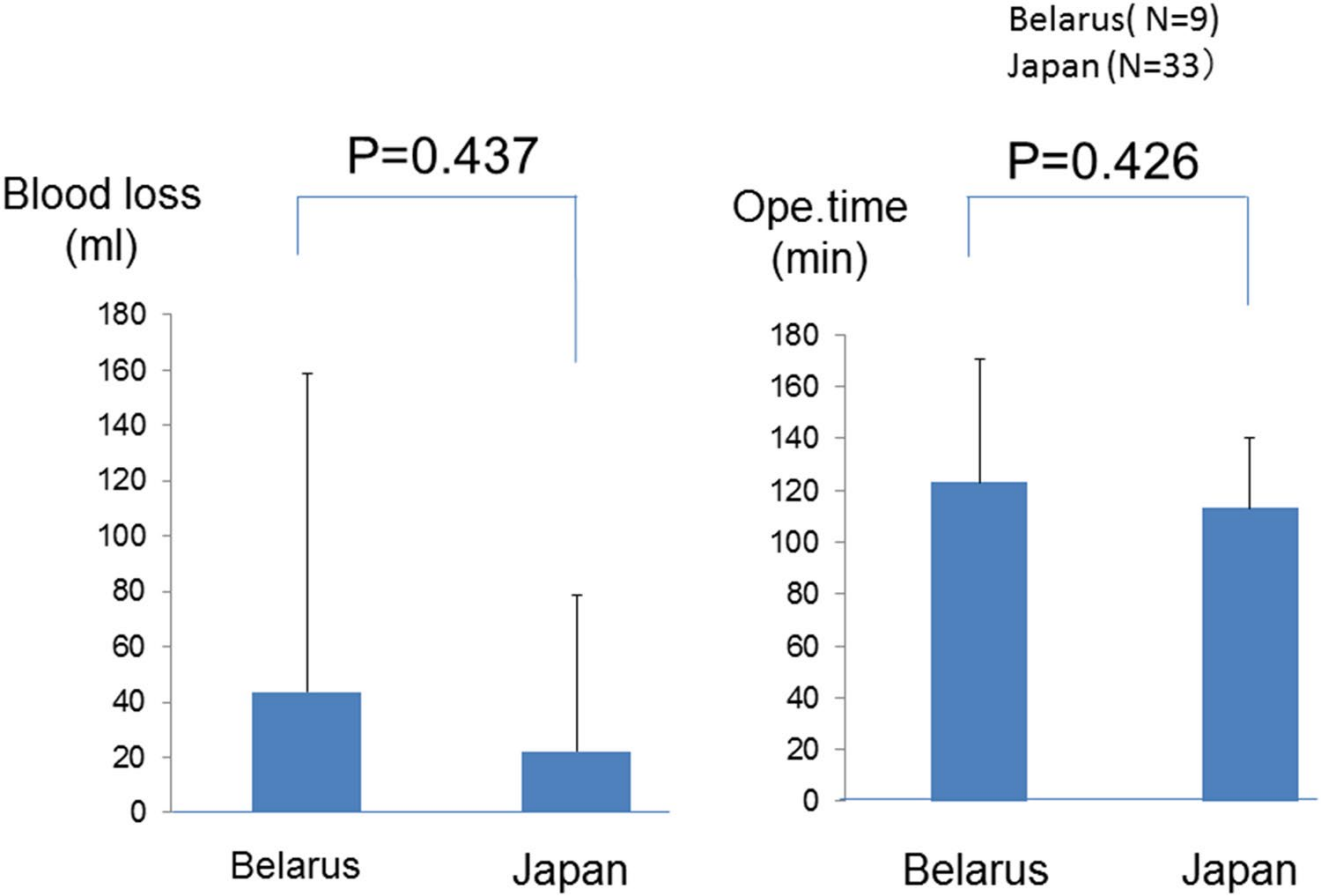
Statistical analysis of blood loss and operating times of the VANS method in Belarus and Japan. There was no significant difference between these factors in both countries when performed by a single surgeon. The VANS method appeared to be practical, simple, and feasible, with cosmetic advantages

This study was carried out simply for our interest. We do not understand the Belarusian language and most people in Belarus do not speak English or Japanese, although an interpreter was in the operating room. Moreover, the operative equipment and hospital environments were different. Under these circumstances, a surgeon performed the VANS method for nine Belarusian patients in Belarus, and we compared the operating time and blood loss with those of 33 VANS procedures performed by the same surgeon in the same period for Japanese patients in Japan. Neither of these factors differed significantly between the groups (Fig. 16) These findings demonstrate that the VANS method is simple, feasible, practical, and safe.

## Discussion

We described various aspects of the VANS method, based on our experience of 935 cases of endoscopic thyroid and parathyroid surgery. First, we described the operative technique in detail. Since the CO_2_ insufflation-related complications of massive emphysema and severe hypercarbia have been resolved by reducing the CO_2_ pressure from 15 to 6–7 mmHg, the VANS method is performed at many locations in Japan and other Asian countries. Notably, according to the JSES questionnaire, 81.5% of the working space is created by the gasless lifting method, which is much simpler and more practical than CO_2_ insufflation, and cost-effective because no CO_2_ insufflation equipment is needed. The underlying endocrine neck diseases and complication rates were similar to those revealed in the questionnaire by JSES, except for skin burn and emphysema, although the exact number of these complications was relatively low. Since the recent approval by the Japanese government for insurance coverage of endoscopic thyroid surgery for thyroid and parathyroid diseases, abstracts in the three major congresses for endoscopic thyroid surgery are expected to increase.

Based on our findings, doctors will probably need to perform at least 30 procedures to master the VANS method and reduce the operating time and blood loss. Moreover, tumors smaller than 5 cm in diameter are associated with shorter operating times and less blood loss. Interestingly, discomfort in the neck was higher in the 10–19, 40–59, and over 70 age groups. Pain can be expressed accurately and the level of pain experienced in the first year after surgery varied from patient to patient, although it was lower in the VANS group than in the conventional group within 6 months of surgery, as expected.

Finally, we asked patients who underwent conventional surgery, “Would you have selected endoscopic thyroid surgery if it had existed at the time of your operation?” This question was asked prior to the government approving insurance coverage for this surgery. Only 60% of these patients answered that they would have selected the VANS method, which was lower than our expectation. When investigating the patients’ satisfaction with wound outcomes after the VANS method versus conventional surgery, we should have enabled patients to compare their outcomes with those of patients undergoing the other procedure.

Recently, intraoperative nerve monitoring [38–41], a transoral approach [42–46], and robotic surgery [47–50] have emerged in endoscopic thyroid surgery. We believe that intraoperative nerve monitoring is efficient for identifying and preserving the recurrent nerve and external branch of the superior laryngeal nerve. However, it will take time for transoral endoscopic thyroidectomy to become established because of the peculiar angle used, and for robotic surgery to become cost-effective, feasible, and practical for endoscopic thyroid surgery in Japan.

## Conclusion

The VANS method is a feasible, practical, safe, and inexpensive procedure with cosmetic advantages over conventional surgery.
